# Feasibility Analysis of Phenotype Quantification from Unstructured Clinical Interactions

**DOI:** 10.5334/cpsy.78

**Published:** 2022-01-11

**Authors:** Daniel S. Barron, Stephen Heisig, Carla Agurto, Raquel Norel, Brittany Quagan, Albert Powers, Michael L. Birnbaum, Todd Constable, Guillermo Cecchi, John H. Krystal

**Affiliations:** 1 Department of Psychiatry, Yale University, New Haven, CT, USA; 2 Department of Psychiatry, Brigham and Women’s Hospital, Harvard Medical School, Boston, MA, USA; 3 Department of Anesthesiology and Pain Medicine, Brigham and Women’s Hospital, Harvard Medical School, Boston, MA, USA; 4 Department of Neurology, Icahn School of Medicine, Mt. Sinai, NY, USA; 5 T.J. Watson IBM Research Laboratory, Yorktown Heights, NY, USA; 6 Department of Psychiatric Research, The Zucker Hillside Hospital, Northwell Health, Glen Oaks, NY, USA; 7 Department of Radiology and Biomedical Imaging, Yale School of Medicine, New Haven, CT, USA; 8 Department of Neurosurgery, Yale School of Medicine, New Haven, CT 06520, USA

**Keywords:** digital phenotype, conversation, voice, facial feature, acoustic

## Abstract

We conducted a feasibility analysis to determine the quality of data that could be collected ambiently during routine clinical conversations. We used inexpensive, consumer-grade hardware to record unstructured dialogue and open-source software tools to quantify and model face, voice (acoustic and language) and movement features. We used an external validation set to perform proof-of-concept predictive analyses and show that clinically relevant measures can be produced without a restrictive protocol.

Clinicians evaluate a patient’s mental state by how (i.e., vocal prosody, facial expression) and what (i.e., speech content) they say during clinical interaction. This routine conversation produces a wealth of potentially useful data that currently are neither recorded nor quantitatively analyzed, representing a missed opportunity to define more precise phenotypes that could offer value to personalized, precise treatment indications and outcome measures in mental healthcare.

Clinical scales (e.g., the Brief Psychiatric Rating Scale (BPRS) (Overall & Gorham, 1962)) attempt to standardize a clinician’s observations, however they suffer from subjectivity, inter-rater variability, forced phenotypic dimensionality and lack of granularity. Manual, qualitative measures of facial expression date back at least to a 1964 study in which patients admitted for depression were video recorded and their facial action units (AUs) were coded (Ekman, 1964) Manual coding remains labor intensive, time-consuming and expensive (Cohn et al., 2007) limiting its clinical utility.

There is a long history of research analyzing verbal and nonverbal behavior to assess clinical status in psychiatry (Mahl et al., 1954; Mahl, 1964). Automated, quantitative verbal and nonverbal behavioral measures have flourished in the last decade. Objective measures of speech (de Jong & Wempe, 2009) and facial expression (Baltrusaitis et al., 2018) can be generated from video-recordings with high fidelity and accuracy. Automated measures derived from structured interviews have been correlated with psychiatric symptoms or clinical scales relevant to PTSD, depression, schizophrenia, or substance use (Bishay et al., 2019; Cohn et al., 2009; Stratou et al., 2015; Agurto et al., 2019). However, because such studies report protocols that involve lengthy structured interviews conducted by trained research raters and require expensive or bulky recording equipment, they are not conveniently or clinically deployable.

We describe a clinically deployable framework for gathering and automatically quantifying behavioral health data that are already generated during standard, unstructured clinical conversations. We used inexpensive, consumer-grade equipment (a Zoom q8 video recorder, $350, and Sennheiser AVX-ME2 SET Digital Camera-Mount Wireless Omni Lavalier Microphone System, $699) and publicly available, automated tools to derive face, acoustic, and linguistic measures. We reference clinician-ratings standardized in the BPRS to show that measures derived from unstructured conversations infer clinically relevant phenotypic information about disease state within sample and in an external validation set.

Patient data across 48 sessions in 8 acutely ill inpatients voluntarily recruited from the local emergency department were recorded daily from admission to discharge at the CT Mental Health Center (CMHC’s) Clinical Neuroscience Research Unit (CNRU) from June 2019 to February 2020 under an IRB approved by Yale University (HIC#2000025490). Open-ended questions were used to elicit natural, nonstructured spoken responses. An independent validation dataset comprising 142 structured sessions in 81 patients was collected at The Zucker Hillside Hospital at Northwell Health in the outpatient setting from September 2018 and July 2019 under an IRB approved by Northwell Health (IRB#18-0137). See Supplementary Materials for demographic data.

Video files were processed with the Openface facial behavior analysis toolkit (Baltrusaitis et al., 2018) to estimate face actions units (Ekman, 2013; Ekman et al., 1969), gaze, and head pose features. These features, obtained for each frame in a time-series are summarized using several descriptors (e.g. mean) and were analyzed per-session from inpatient admission to discharge to quantify a patient’s longitudinal treatment trajectory. ***Figure 1A*** illustrates that mean head and gaze velocity (simple measures of psychomotor activity) recapitulate the BPRS depression inventory from inpatient admission to discharge. We further show these longitudinal trajectories within the context of a larger, independently collected dataset.

**Figure 1:**
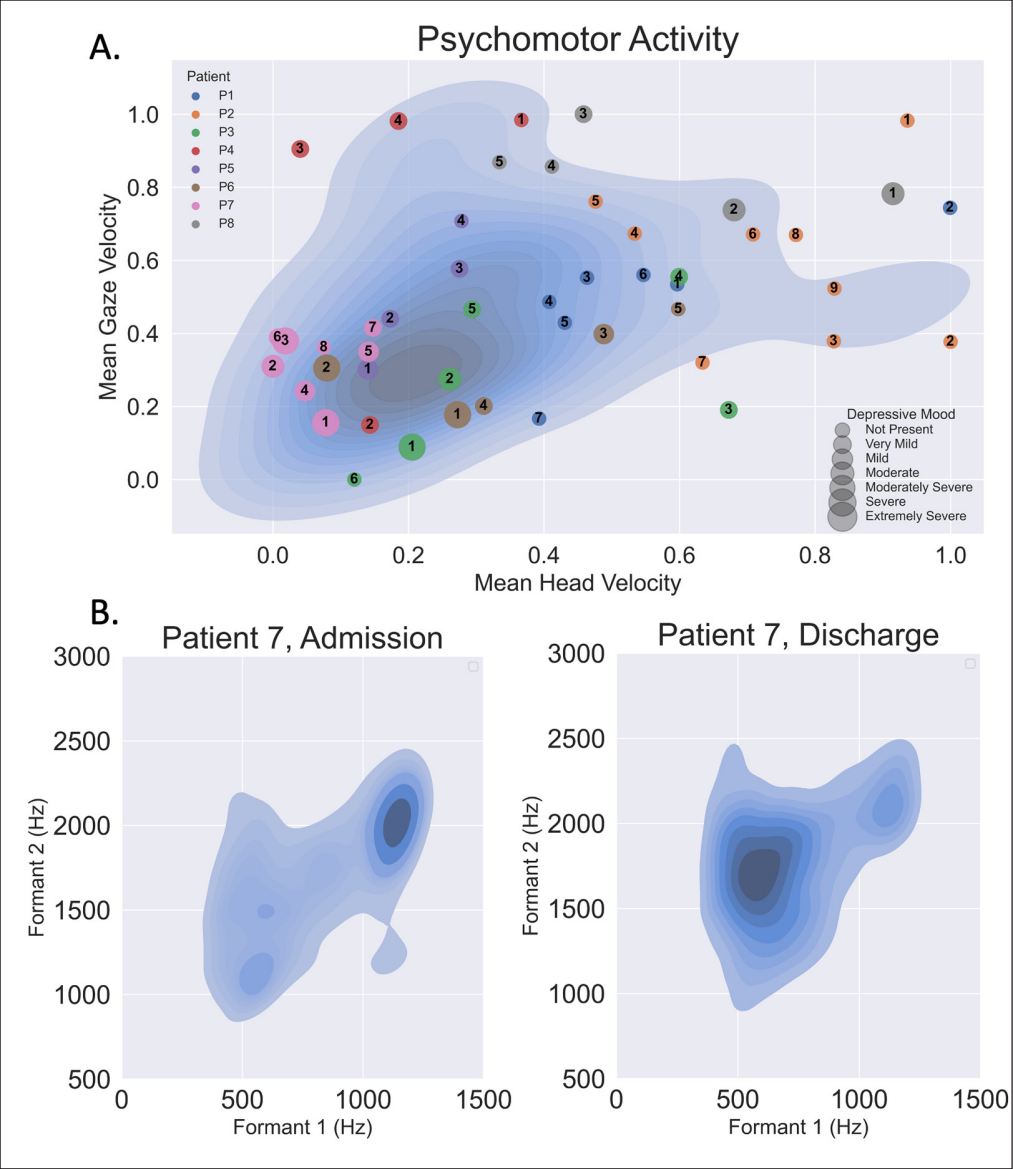
**Quantitative face and voice features versus clinical progress. (A)** Face psychomotor activity (gaze and head pose in radians per second scaled from 0 to 1) sized by BPRS depression score. The non-anxious depressed patients (3,5,6,7) tended to have more movement as their depressive mood scores increased and less overall than non-depressed patients. Patient 8 was anxious and depressed, hence her head movement decreased as she recovered. Individual patients are assigned their own color and are numbered by session (Patient 4’s third session is represented by a red dot with the number 3). The background density plot (blue hues) provides context from a larger (142 sessions), independently collected dataset, illustrating how features derived from unstructured conversation fall within the scope of a structured exam. **(B)** Vowel space density plots visually reveal the trajectory of acoustic changes in a depressed patient who received ketamine infusion. Reduced vowel space is clearly visible during an early session (representing a more monotonic voice, left) compared with a later session (representing a more varied voice, right) for the same patient. The BPRS depressive mood score for the first session was 6 and 0 for the second. Restricted vowel space is a well-documented acoustic feature which has been shown to correlate with depressive mood (Scherer et al., 2016).

Audio recordings were processed to characterize acoustic features such as vowel space and speech rate. ***Figure 1B*** demonstrates how a depressed patient’s vowel space, or the area subtended by the frequency range of the first two formants (the first two resonant frequencies of the vocal track) here illustrated by the kernel density estimate of the frequencies of the first two formants quantifies the “flattened affect” and “slowed, monotonous speech” phenomena and shows how the vowel space becomes more diffuse and varied with clinical improvement related to ketamine treatment (the only patient to receive ketamine).

We transcribed all recordings and extracted psycholinguistic categories using Linguistic Inquiry and Word Count (LIWC). ***Figure 2A*** illustrates speech structure analysis wherein the number of words produced by the patient relative to the therapist quantify a patient’s conversational engagement. We further show (***Figure 2B***) how the measured perseveration of a manic patient’s grandiose delusion (in this case, being a world-class consultant) disappears with clinical improvement. Semantic similarity was calculated as the cosine similarity between the vector for the word ‘consulting’ and the text in each interview using Global Vectors for Word Representation (GloVe) embeddings (Pennington et al., 2014).

**Figure 2:**
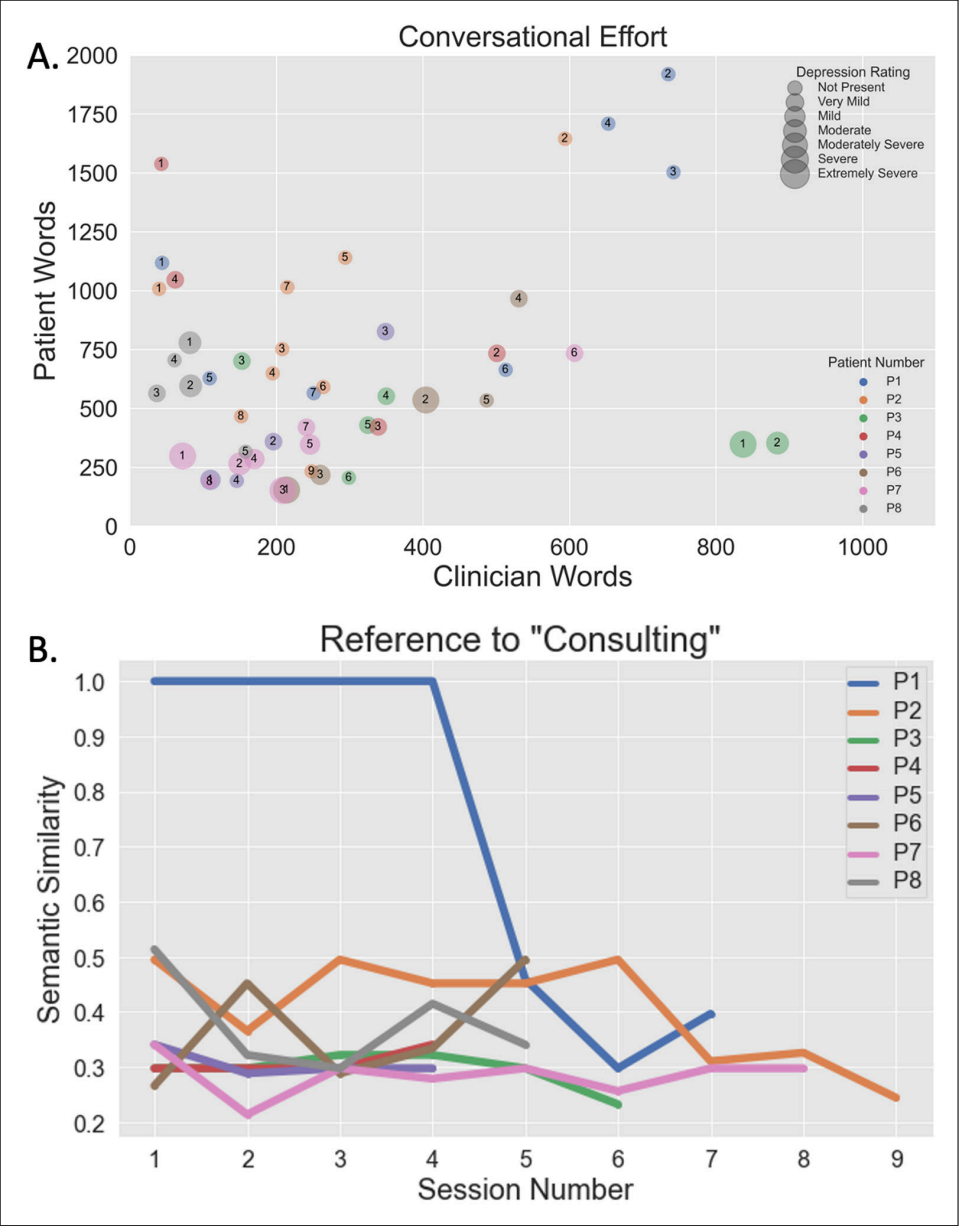
**Conversational effort and speech content can be measured from unstructured clinical conversation. A)** Conversational effort illustrates words per session for clinician and patient. The Patient 8 (grey circles) displays an anxious depression phenotype producing markedly more words than the clinician. Participant feature data plotted longitudinally exposes subtle changes in objective measures that, we speculate, the human brain would find difficult to identify from memory. The cross-sectional plot facilitates patient comparison. **B)** Speech content analysis quantifies diminution of perseveration. Here, we used semantic analysis to calculate the cosine distance between the single perseverating patient’s speech vectors to the GloVe vector for the concept “consulting.” As the patient’s perseveration decreased, this topic became less frequent. No other patients displayed this behavior.

Our proof-of-concept predictive analyses suggested that non-structured clinical interviews have sufficient signal to motivate future model development. Given the high likelihood of overfitting, all within-sample analyses are considered exploratory notwithstanding their statistical significance (see ***Table 1***). Out-of-sample analyses of the external, Northwell dataset showed that facial features were able to predict BPRS subscore for blunted affect, however the models did not perform as well when predicting depressed mood.

**Table 1:** **Proof-of-concept predictive analyses indicate nonstructured interviews have sufficient signal to merit future model development.** Results are reported for acoustic, facial, and linguistic feature types both within-sample for our internal dataset collected at the CNRU and out-of-sample for an external dataset collected independently by Northwell Health. Given the high likelihood of overfitting, within-sample analyses are considered exploratory. In the out-of-sample analyses of the external dataset, facial features were able to predict BPRS subscore for Blunted Affect, however the models did not perform as well when predicting Depressed Mood. Given the small sample size relative to the number of features (acoustic = 333, facial = 1030, linguistic = 24), prediction performance is reported as a Spearman rank coefficient and p-value. Regression algorithms used to obtain the best result noted as LR-linear, RI-ridge, LA-lasso, SV-support vector.


	FEATURES	INPATIENT CNRU STUDY (N = 8, 48 SESSIONS) WITHIN-SAMPLE ANALYSES	INDEPENDENT TESTING: NORTHWELL HEALTH DATASET (N = 81, 142 SESSIONS) OUT-OF-SAMPLE ANALYSES

BPRS Blunted Affect	Acoustic	0.26, p = 7E-2, SV	0.35, p = 4E-5, LA

	Facial	0.72, p < 1E-5, SV	0.30, p = 5E-4, RI

	Linguistic	0.72, p < 1E-5, SV	–

BPRS Depressive Mood	Acoustic	0.36, p = 1E-2, RI	0.16, p = 7E-2, LR

	Facial	0.63, p < 1E-5, LA	0.12, p = 2E-1, LR

	Linguistic	0.73, p < 1E-5, RI	–


We again note that given the CNRU’s small sample size (48 sessions), we expect overfitting and therefore do not claim a generalizable model on the basis of the within-sample analyses performed on CNRU data alone. Further, it is worth observing that the Northwell data had a higher percentage of African Americans and it is well known that Openface is not optimized for multiple races. Notwithstanding these limitations, we believe our proof-of-concept analyses motivate continued in-clinic data collection and model development.

Previous attempts to quantify conversational behavioral health data have deployed rigid interview protocols, expensive or bulky recording equipment, or costly, specialist ratings that are not feasible or even unsafe in real-life clinical settings or would require drastic changes to the clinical workflow. Our protocol-free approach mines data already produced during routine clinical assessments, thereby only minimally changing the clinical workflow. We show that data gathered on inexpensive, consumer-grade equipment and processed by a suite of automated, open-source analytic tools are of sufficient quality to infer standardized clinician observations. Finally, we demonstrate that the same behavioral phenotypes that trained clinicians perceive as evidence of clinical improvement can be measured and traced over time.

Because our protocol-free approach is flexible to different acuity settings, we believe that clinicians and researchers might incorporate behavioral measurement without the need to redefine their clinical workflow. With further development, our approach might help clinicians passively document known clinically relevant behavior and, in the future, provide parameters for detecting other clinically relevant behaviors that are yet undefined. Though our approach shows promise, it has specific limitations which necessitate discussion:

First, this proof-of-concept study was necessarily limited to correlation with a single—though widely implemented—measure of clinical state: the BPRS. Further research will evaluate whether overall symptom domains might be made more precise (e.g., “pressured” or “rapid” speech might be quantitatively operationalized in terms of speech rate or articulation rate) or whether aspects of other clinical scales (van Steenbergen-Weijenburg et al., 2010) might be automated. The long-term value of behavioral quantification very well could be identifying features of behavior that the human brain cannot detect such as microexpressivity, subtle changes in speech coherence (Bedi et al., 2015), or the use of specific linguistic elements (e.g., determiners (Corcoran et al., 2018)) which have previously been associated with clinical conditions. Accumulating a database of these features across diagnoses and confounding factors will be necessary to build models with sufficient predictive power to be clinically useful.

Second, our proof-of-concept analyses focused on capturing and predicting behavioral signals of known clinical relevance; i.e., we quantified aspects of clinical conversation that are already widely believed to have clinical value. While documenting behavioral measurements represents an improvement over standard, language-based practices, we believe that the real value of behavioral quantification will lie in discovering new, previously undetected signals that might have implications for treatment and outcome. These analyses will require further research and are beyond the scope of this brief communication.

In summary, we demonstrate that clinical conversation—even when brief and unstructured—offers a currently untapped source of data that can be gathered with low-cost tools in a clinically feasible manner. We demonstrate and openly release code for our automated pipeline, which shows that conversational measures capture clinically relevant phenotypic information.

## Additional File

The additional file for this article can be found as follows:

10.5334/cpsy.78.s1 Online Supplementary Material. Further description of analytic pipeline and patient demographic information.
