# Supplementary material for: Feasibility Analysis of Phenotype Quantification from Unstructured Clinical Interactions
Source: Comput Psychiatr. 2022 Jan 11;6(1):1–7. doi: 10.5334/cpsy.78 (PMC11104416; doi:10.5334/cpsy.78)
Supplement: Online Supplementary Material. — Further description of analytic pipeline and patient demographic information. [file cpsy-6-1-78-s1.pdf]

Online Supplementary Material

Methods

Patient Populations

We analyzed data from two different studies. All participants were voluntarily recruited from July 2019 to February 2020 under an IRB approved by Yale University (HIC#2000025490). Acutely ill inpatients were recruited from the Yale New Haven Hospital's psychiatric emergency room and were admitted to the CMHC's CNRU following an explanation of and voluntary consent to the study design. While at the CMHC, they received standard-of-care treatment that was in no way dictated by the study. From admission to discharge, participants met daily with a clinician (DSB), who engaged in ~3 minute unstructured conversation each day. Participants did not receive financial compensation for their participation, however they were not billed by the CMHC for their inpatient psychiatric hospitalization. Recruitment stopped because of the pandemic, limiting sample size.

An external validation dataset was independently collected at the Zucker Hillside Hospital's (Northwell Health) inpatient and outpatient psychiatric clinics, as part of an observational study. Participants 15- 35 years old diagnosed with schizophrenia spectrum disorder (SSD) or bipolar disorder (BD) were recruited between September 2018 and July 2019 under an IRB approved by Northwell Health (IRB#18-0137). All data were obtained in a non-identifiable format. Study demographics can be referenced in Supplementary Table 1.

Audiovisual Recording Equipment

CNRU video data were collected with a Zoom 8 video recorder ($350), which has two audio inputs and built-in pre-amplifier. Audio data were collected with a Sennheiser AVX-ME2 SET Digital Camera-Mount Wireless Omni Lavalier Microphone System ($699.00) was used for audio recordings. Preprocessing took place on a Lenovo ThinkPad (T470p 2.9GHz Core i7 $2100).

Analytic Pipeline

Video recorded at 30 frames per second was initially processed to extract first-order features using the Openface 2.0 Facial Behavior Analysis Toolkit [1]. This toolkit estimates 18 facial action units [2,3], gaze, and head pose orientation, 68 facial landmarks, as well as the Histogram of Oriented Gradients (HOG) for each frame. Because features are obtained at a resolution of 30Hz (every 0.03s), micro expressions (<0.5 seconds) and macro expressions (0.5-4 seconds) can be detected. To characterize the time series generated by obtaining features at every frame, a python-package called TSFRESH[4] was used. In this way, time series are summarized with several descriptors (e.g., mean, intra-feature correlation and covariance, entropic measures, and distribution parameter estimates). A set of representative features is plotted for human review and quality assurance purposes.

Audio recordings were processed using Praat Phonetics Toolbox[5] to produce acoustic features in three general areas. The first area is pitch and formant features, which describe the speaker's vowel space. These have been correlated with depression and suicidality[6]. The second is related to capture tempo (a prosody component), e.g., pause distribution, speech and articulation rates, and syllable and pulse counts. These are useful in characterizing neurological signs such as psychomotor retardation. The third includes voice quality metrics including jitter,

shimmer, harmonic to noise ratio, mean autocorrelation, and glottal noise excitation ratio which correlate with general health states, medication usage, and emotional state. In addition, we used PyAudioAnalysis[7] library to obtain spectral features, specifically zero-crossing rate, energy, entropy, spectral roll-off, centroids, density, flux, MFCCs, cepstral peak prominence, and chroma values. These types of features have an extensive published history of correlation with neurological states.

Linguistic Analysis

With transcribed speech, basic text characterization and part of speech tagging was performed with the Standford lexparser tagging toolbox.[8] For example, first person pronoun frequency is often altered in depression or other serious health conditions[9]. Linguistic Inquiry and Word Count [10] produces word counts based on topics of interest which are useful to quantify speech content. Finally, to analyze the semantic similarity with respect to concepts, we used GloVe[11] and Google Universal Sentence Embedding[12] at a word and sentence level, respectively.

Proof-of-Concept Predictive Analysis

To preliminarily test whether an unstructured clinical interaction contained sufficient signal to merit future model development, we performed two proof-of-concept experiments: a within-sample, leave-one-subject-out nested cross-validated analysis of the CNRU data (n=8, 48 sessions) and an out-of-sample analysis trained on all CNRU data and tested on the Northwell Health (n=81, 142 sessions) data. Acoustic (n=333), facial (n=1030) and linguistic (n=24) features were normalized and input to common predictive algorithms: linear, ridge, lasso and support vector regression. Given the limited sample size (CNRU patient recruitment was stopped because of the pandemic) and non-gaussian nature of the date, we measured model performance using Spearman rank correlation coefficient given the non-gaussian nature of the data. In the out-of-sample analysis, we did not use linguistic features because the Northwell Health interviews were highly structured, often with yes/no patient responses. We note that given the limited sample size compared to the large number of features, we cannot exclude the possibility of overfitting in the dataset and therefore present these analyses simply as proof-of-concept.

Code Availability: Code to perform the analyses and produce the above figures will be posted as a Jupyter Notebook on GitHub on final acceptance of the paper.

Data availability: While we cannot publicly release the raw video due to participant privacy and security concerns, including HIPAA regulations, we will post the processed, non-identifiable features collected Yale on GitHub on final acceptance of the paper.

Supplementary Table. Demographic information for the three datasets.

Inpatient CNRU Study

	G	Age	Race	Edu	Clinical Diagnosis	Presenting Concern
P1	M	22	W	14	Bipolar 1 Disorder with psychotic features	mania w psychotic features
P2	M	65	W	12	Schizoaffective d/o, alcohol-related dementia	psychosis, off medication
P3	M	19	W	12	OCD, mood disorder w anxiety	CAH with SI
P4	M	43	W	12	Opioid use disorder, mood disorder NOS	suicidal ideation
P5	F	21	A	15	MDD, neurofibromatosis	suicidal ideation
P6	M	20	W	12	MDD	suicidal ideation
P7	F	29	AA	16	MDD, OCD	suicidal ideation
P8	F	28	L	13	PTSD, MDD, GAD	suicide attempt

Northwell Health Validation Dataset

	N	G (M)	Age (mean)	AA/Black	Asian	White	Pacific Islander	Latino	Other
SSD	41	29	23.7	24	6	10	0	5	1
BD	21	7	25.3	35	4	9	0	3	5
HV	27	12	28.5	8	6	10	1	1	2
Total	89	48	25.5	35	16	29	1	9	8

1. Baltrusaitis, T., Zadeh, A., Lim, Y. C. & Morency, L.-P. OpenFace 2.0: Facial Behavior Analysis Toolkit. *2018 13th Ieee Int Conf Automatic Face Gesture Recognit Fg 2018* 59–66 (2018) doi:10.1109/fg.2018.00019.

2. Ekman, P. *Emotion in the Human Face*. (Malor Books Reprint Edition, 2013).

3. Ekman, P., Sorenson, E. R. & Friesen, W. V. Pan-Cultural Elements in Facial Displays of Emotion. *Science* 164, 86–88 (1969).

4. Christ, M., Braun, N., Neuffer, J. & Kempa-Liehr, A. W. Time Series FeatuRe Extraction on basis of Scalable Hypothesis tests (tsfresh – A Python package). *Neurocomputing* 307, 72–77 (2001).

5. Boersma, P. & Heuven, V. V. Speak and unSpeak with PRAAT. *Glot International* 5, 341–347 (2001).

6. France, D. J., Shiavi, R. G., Silverman, S., Silverman, M. & Wilkes, M. Acoustical properties of speech as indicators of depression and suicidal risk. *Ieee T Bio-med Eng* 47, 829–837 (2000).

7. Giannakopoulos, T. pyAudioAnalysis: An Open-Source Python Library for Audio Signal Analysis. *Plos One* 10, e0144610 (2015).

8. Klein, D. & Manning, C. D. Fast Exact Inference with a Factored Model for Natural Language Parsing. *Advances in Neural Information Processing Systems 15* (2002).

9. Corcoran, C. M. *et al.* Prediction of psychosis across protocols and risk cohorts using automated language analysis. *World Psychiatry* 17, 67–75 (2018).

10. Pennebaker, J. W., Boyd, R. L., Jordan, K. & Blackburn, K. The development and psychometric properties of LIWC2015. doi:10.15781/t29g6z.

11. Pennington, J., Socher, R. & Manning, C. Glove: Global Vectors for Word Representation. *Proceedings of the 2014 Conference on Empirical Methods in Natural Language Processing* 1532–1543 (2014) doi:10.3115/v1/d14-1162.

12. Cer, D. *et al.* Universal Sentence Encoder. *Arxiv* (2018).
